# Challenges in Antibody Development against Tn and Sialyl-Tn Antigens

**DOI:** 10.3390/biom5031783

**Published:** 2015-08-11

**Authors:** Liliana R. Loureiro, Mylène A. Carrascal, Ana Barbas, José S. Ramalho, Carlos Novo, Philippe Delannoy, Paula A. Videira

**Affiliations:** 1 CEDOC, Chronic Diseases Research Center, NOVA Medical School/Faculdade de Ciências Médicas, Universidade NOVA de Lisboa, Campo dos Mártires da Pátria, 130, Lisboa 1169-056, Portugal; E-Mails: liliana.loureiro@fcm.unl.pt (L.R.L.); mylene.carrascal@fcm.unl.pt (M.A.C.); jose.ramalho@fcm.unl.pt (J.S.R.); cnovo@ihmt.unl.pt (C.N.); 2 IBET—Instituto de Biologia Experimental e Tecnológica, Apartado 12, Oeiras 2781-901, Portugal; E-Mail: ab@ibet.pt; 3 IHMT, Instituto de Higiene e Medicina Tropical, Universidade NOVA de Lisboa, Rua da Junqueira 100, Lisboa 1349-008, Portugal; 4 Structural and Functional Glycobiology Unit, UMR CNRS 8576, University of Lille, Villeneuve d’Ascq 59655, France; E-Mail: philippe.delannoy@univ-lille1.fr; 5 Departamento Ciências da Vida, Faculdade de Ciências e Tecnologia, Universidade NOVA de Lisboa, Caparica 2829-516, Portugal

**Keywords:** Tn antigen, Sialyl Tn antigen, immune response, therapeutic antibodies, antibody production

## Abstract

The carbohydrate antigens Tn and sialyl-Tn (STn) are expressed in most carcinomas and usually absent in healthy tissues. These antigens have been correlated with cancer progression and poor prognosis, and associated with immunosuppressive microenvironment. Presently they are used in clinical trials as therapeutic vaccination, but with limited success due to their low immunogenicity. Alternatively, anti-Tn and/or STn antibodies may be used to harness the immune system against tumor cells. Whilst the development of antibodies against these antigens had a boost two decades ago for diagnostic use, so far no such antibody entered into clinical trials. Possible limitations are the low specificity and efficiency of existing antibodies and that novel antibodies are still necessary. The vast array of methodologies available today will allow rapid antibody development and novel formats. Following the advent of hybridoma technology, the immortalization of human B cells became a methodology to obtain human monoclonal antibodies with better specificity. Advances in molecular biology including phage display technology for high throughput screening, transgenic mice and more recently molecularly engineered antibodies enhanced the field of antibody production. The development of novel antibodies against Tn and STn taking advantage of innovative technologies and engineering techniques may result in innovative therapeutic antibodies for cancer treatment.

## 1. Introduction

The deranged expression of glycans is a hallmark of cancer. Glycans such as the Thomsen-Friedenreich related antigens, Tn and sialyl-Tn (STn), show a very tumor specific pattern and, in most cancers, they are associated with disease progression and patient’s response to treatment. These antigens have therefore triggered enthusiasm among the scientific community as diagnostic and prognostic biomarkers. A relevant number of antibodies were developed and characterized to be used in immunohistochemistry staining protocols. Yet the added value of the identification of STn or Tn as biomarkers has proven insufficient, compared to standard protocols, to justify its regular use in clinical practice. A second and more pronounced enthusiasm arose with the development of immunotherapies targeting these antigens to boost immune responses, *i.e.*, vaccination. Nevertheless, the enthusiasm was rapidly refrained due to the unsuccessful results from clinical trials. More recent findings have helped to understand that these antigens have potential to dampen immune responses which oblige us to revise the efficacy of immunization procedures and to search for more effective strategies for treatment of tumors expressing these antigens.

Interestingly, the antibodies developed against these antigens have been mainly regarded as tools for diagnosis and prognosis. The exploitation of these antibodies as immunotherapeutic tools to elicit anti-tumor immune responses has been completely disregarded. Here we revise the efforts built towards the development of high affinity antibodies against STn and Tn antigens and the methodologies involved. A portfolio of cutting edge methodology is here described that can be used to speed the production of more effective antibodies or novel engineered antibody-based molecules as innovative immunotherapies against STn or Tn.

## 2. Glycosylation in Cancer

Glycosylation is the most frequent and well-known posttranslational modification of proteins. Glycans are present in all living organisms and regulate a diversity of biological processes, including protein folding and intracellular trafficking, cell-cell and cell-matrix interactions, cellular differentiations and the immune response [1,2]. The relevance of glycosylation is highlighted by the fact that approximately 1%–2% of human genes are required for glycan biosynthesis, which is catalyzed by the enzymatic reaction of several enzymes that transfer mono or oligosaccharides, *i.e.*, glycosyltransferases, with their strict specificity for both donor and acceptor substrates [3].

In humans, proteins can be glycosylated with two main types of glycans: *N*-glycans and *O*-glycans. *N*-glycans are covalently linked via an amide bond to asparagine in the polypeptide sequence, and it usually involves the Asn-X-Ser/Thr (X ≠ Pro) sequence. *O*-glycans are often covalently linked to the hydroxyl group of a serine or threonine into the polypeptide via *N*-acetylgalactosamine (GalNAc) [4]. As glycan biosynthesis is not a template-based process such as DNA, RNA, or protein synthesis, glycan expression depends on the balance achieved by the expression and activity levels of the different enzymes involved in the glycosylation process, as well as by their localization/organization, for instance in the Endoplasmic Reticulum and Golgi apparatus, and on the availability of precursor monosaccharide molecules [5].

In the mid-1990s there was a peak of interest in glycosylation because a significant correlation between certain types of altered glycosylation and cancer prognosis was observed. In fact, aberrant glycosylation frequently occurs in cancer and plays a pivotal role in cancer progression, angiogenesis and metastasis, cell-cell contact, motility and epithelial-mesenchymal transition (EMT) in cancer cells. These alterations of glycosylation comprises under- or over-expression of glycan structures, as well as the appearance of novel or truncated structures. However, despite the diversity of glycans at the cell surface, only a few distinct structures are associated with malignant transformation and tumor progression. This suggests that specific glycosylation patterns contribute to precise mechanisms such as gain of function, cell fitness and survival [6].

**Figure 1:**
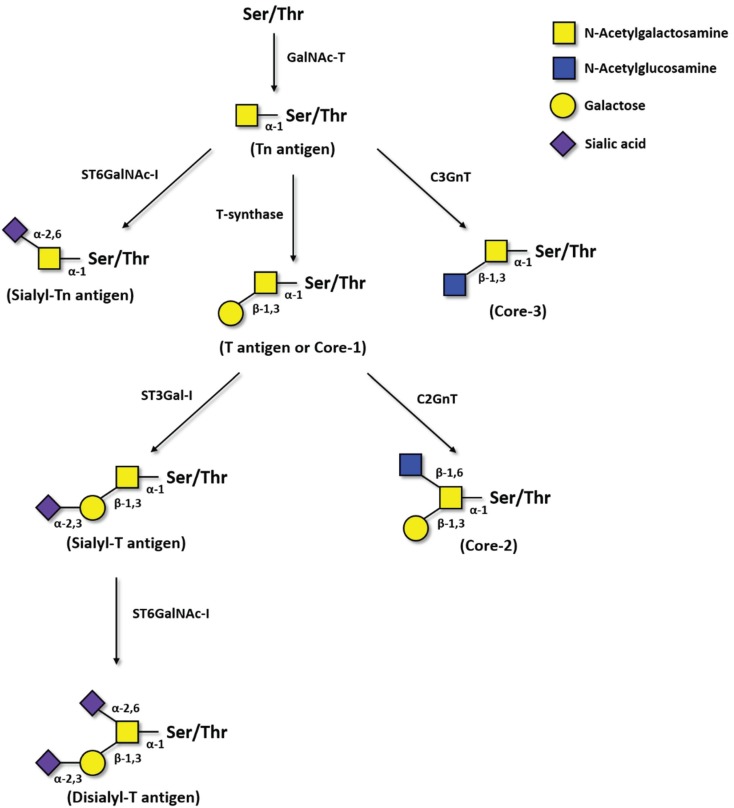
Pathways of Thomsen-Friedenreich antigens biosynthesis. First, GalNAc is transferred to a serine or threonine residue in a polypeptide by peptidyl-*N*-acetylgalactosaminyltransferases (GalNAc-T). GalNAcα1-Ser/Thr, *i.e.*, the Tn antigen, is then converted by T-synthase to Galβ1-3GalNAcα1-Ser/Thr, *i.e.*, the T antigen or core-1 structure. The Tn antigen can be also sialylated by ST6GalNAc-I forming Neu5Acα2-6GalNAcα1-Ser/Thr, *i.e.*, the sialyl-Tn. GalNAcα1-Ser/Thr can also be converted to core-3 structures by the β3GnT-6. T antigen is converted to core-2 structures by C2GnT-1, -2, and -3 or can be sialylated by ST3Gal-I, originating the sialyl-T antigen. Moreover, sialyl-T antigen can be sialylated by ST6GalNAc-I originating disialyl-T antigen.

One of the most common aberrant glycosylation in cancer is the neo-expression of Thomsen-Friedenreich-related antigens. This family of incomplete *O*-glycans (Figure 1) includes: The Thomsen-nouveau (Tn) antigen (GalNAc-α1-*O*-Ser/Thr), which consists of one residue of GalNAc alpha-*O*-linked to a serine or a threonine residue in the polypeptide chain; the Thomsen-Friedenreich (T) antigen (Galβ1-3GalNAc-α1-*O*-Ser/Thr) or unmodified core-1 structure, where a residue of galactose (Gal) is β-linked to GalNAc; the STn antigen, in which the GalNAc residue on Tn antigen is bound to a sialic acid (Neu5Ac) on carbon 6; and the sialyl-T (ST), in which the Gal residue on T antigen is bound to a Neu5Ac on carbon 3 (Figure 1). The sialylation of both Tn and T antigens blocks the further elongation of core-1 structures, with the exception of ST (*i.e.*, monosialyl-T) that can still be converted into disialyl-T structure.

Historically, the T antigen was first identified in 1930 by Thomsen, in collaboration with Friedenreich, in a blood sample contaminated by bacteria. Specifically, the action of bacterial neuraminidase released the sialic acid that covered the glycan chain, exposing the T antigen on red blood cells, which were then recognized by anti-T antibodies present in the sera, causing the subsequent hemagglutination [7]. The expression of Tn and STn antigens were later discovered in 1957 by Moreau on subpopulations of blood cells characterizing a rare hematological disorder, the Tn syndrome [8].

The Thomsen-Friedenreich family antigens have been initially called as oncofetal antigens as they are generally precursors in normal complex *O*-glycan chains [9]. Mainly expressed on epithelial cells, the overexpression of these antigens has been reported in several different human tumors, usually due to defects in the secretory pathway organelles (Endoplasmic Reticulum and Golgi) and to altered glycosyltransferase expression [6].

T antigen is overexpressed in breast, colon, pancreas and lung cancer; but it can also be detected in up to 40% of normal tissues [4] and in inflamed tissues. Similarly, the ST expression is largely increased in some cancers when compared to normal tissues, such as breast and bladder cancer [10,11,12], but also expressed in normal epithelium, many serum proteins and leukocytes [13,14]. In contrast, the Tn and the STn antigens show a better cancer associated pattern, with absent or very limited expression in adult healthy tissues [15,16,17], and are therefore more interesting as therapeutic targets.

### 2.1. Tn Antigen

Tn antigen is a pan-carcinoma antigen, expressed on a majority of carcinomas, such as breast, pancreas, colon, lung and bladder, being less common in hematological malignancies. In normal tissues, the Tn is only expressed in embryonic brain [18] and is shielded in adult tissue. Tn is actually a normal biosynthetic precursor of *O*-glycans however not a normal terminal product, being occasionally observed in adult normal cells in the secretory apparatus, but not found on proteins at cell surface or secreted [4,15].

Tn is normally extended by the β1,3-galactosyltransferase C1GalT1, also termed T-synthase or core-1 synthase, or by the core-3 β1,3-*N*-acetylglucosaminetransferase (C3GnT) to form T antigen (core-1) or core-3 structure, respectively (Figure 1). In cancer, the loss of T-synthase or C3GnT enzymes may lead to overexpression of Tn antigen [19,20]. Altered expression of GalNAc-Ts glycosyltransferases, and re-localization from the Golgi to the endoplasmic reticulum, also results in Tn overexpression. In addition, defects in Cosmc, a chaperone essential for the activity of C1GalT1, due to epigenetic silencing or mutations can also lead to increase expression of Tn antigen in cancer [21,22].

Overexpression of Tn antigens promotes cancer cell proliferation and invasiveness. In fact, in breast cancer, the accumulation of Tn-bearing proteins in lamellipodia at the cell surface promotes cell adhesion, cell motility and invasiveness [23]. Strikingly, the Tn antigen is also detected at early stages of tumor development and may serve as a biomarker, since its expression is associated with invasive and highly proliferative tumors, and metastasis [24].

### 2.2. Sialyl-Tn Antigen

Sialyl-Tn is also a pan-carcinoma antigen that is expressed early in tumorigenesis. In contrast to Tn, STn is not a normal biosynthetic precursor, meaning that its expression is necessarily pathologic [4]. As a side note, in some normal tissues, such as colon, the sialic acid residue of STn may be *O*-acetylated, thus masking the STn and therefore its recognition by anti-STn antibodies [25]. No other sugars are known to be added to the STn antigen. STn is often co-expressed with Tn and therefore mechanisms that result in Tn overexpression likely apply to STn. Specific mechanisms for STn overexpression, include ST6GalNAc-I upregulation, or re-localization from the Golgi to the endoplasmic reticulum and also loss of *O*-acetyl groups from STn, have been demonstrated in some cancers [26,27,28,29].

Specifically, STn expression modulate a malignant phenotype inducing a more aggressive cell behavior in gastric and breast carcinoma cells, such as decreased cell-cell aggregation and increased extracellular matrix adhesion, migration and invasion [30,31,32,33].

### 2.3. Pathological Role of Tn and STn in Cancer

Both Tn and STn antigens have been correlated with cancer progression and poor survival. High expression of Tn and STn in human ovarian carcinoma was associated with disease stage, histological grade and low overall survival time [34]. In addition, Tn has been shown as good biomarker of invasive cervix carcinoma [35]. Moreover, STn expression was associated with invasiveness of liver cancer [36] and with lower survival rate in advanced gastric carcinomas [37]. In breast cancer, STn expression was associated with lymph node metastasis and high histological grade [38], as well as with resistance to adjuvant chemotherapy [39]. STn antigen was also related to high grade tumors, elevated proliferation rates and high risk of recurrence and progression in bladder tumors [29].

The combination of both features: An acceptable tumor specificity and almost absence of expression of Tn and STn in normal cells; together with their association to tumor prognosis and higher malignancy, including metastization, turned Tn and STn antigens as excellent targets to be used in therapeutics.

## 3. Immune Response and Immunotherapies against Carbohydrates

The immune response is key not only to elicit protection against pathogens, but also to maintain immune surveillance against the development of malignant cells. From this perspective, the development of cancer can be seen as a failure of immune surveillance [40]. Most tumor associated carbohydrates do not elicit strong humoral responses and, in fact, evidences have shown that their aberrant expression is one of the mechanisms developed by cancer cells to prevent effective immune responses against cancer [5].

The mechanisms to provide anti-tumor protection can be broadly categorized into the cellular and the humoral immune responses. While cellular immune response involve mainly cytotoxic lymphocytes such as cytotoxic T cells that may potentially eliminate tumor cells; the humoral response involves B cells and the production of antibodies against specific tumor antigens, that ultimately lead to antibody dependent cytotoxicity, as mentioned in more detail below. Both arms of the immune response involve the activation of a variety of other immune cells, which ultimately converge for both responses. For the elicitation of cellular immune responses, antigen presenting cells such as dendritic cells (DCs), macrophages and B cells, have to uptake antigens and then present through the major histocompatibility complex (MHC) a small portion of peptide antigens (epitopes) to activate both specific CD8^+^ cytotoxic T cells and CD4^+^ helper T (Th) cells. The receptors of both cytotoxic and Th cells, *i.e.*, T cell receptors (TCR), are restricted to recognize peptides, presented in the context of MHC class I or class II, respectively, by the antigen presenting cells. The cellular immune response is therefore mainly formatted to fight peptide antigens and this may impose a limitation to develop strong immune responses against carbohydrates. While it has been reported that cytotoxic T cells may recognize mono- and disaccharides attached to peptides with Ser or Thr [41,42,43,44,45], further investigations are necessary to better understand the relevance of such recognition in the context of antitumor immune responses.

**Figure 2:**
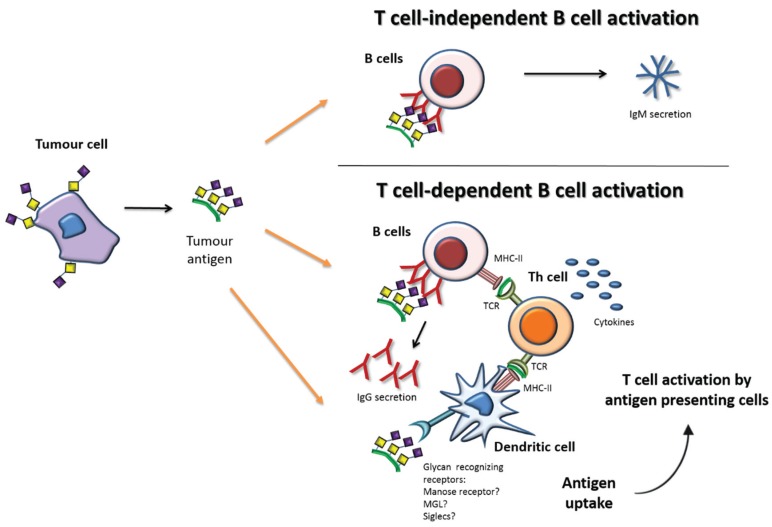
Simplified depiction of tumor antigen recognition and the induction of humoral immune responses. Upper panel—Glycan antigens can be directly recognized by B cells and the cross-linking of the B cell receptors lead to IgM secretion through T cell-independent B cell activation. Lower panel—Tumor antigens can be endocytosed by antigen presenting cells, such as dendritic cells, or B cells and then presented to helper T cells (Th) cells thus leading to T cell activation and subsequent T cell-dependent B cell activation. Adapted from [46].

The humoral immune response against carbohydrates plays a key role in antitumor responses. In contrast to TCR, the B cell receptors (BCR) can recognize directly carbohydrate antigens with no need for presentation through MHC. Cross-linking of the antigen on BCR activates B cells, which initiate the secretion of IgM antibodies. IgM is the first immunoglobulin isotype to be secreted by plasma B cells (effector B cells), characterized by a pentameric structure with relatively low affinity to antigens and short time period, as compared with the other isotypes. This process is called the T cell-independent B cell activation (Figure 2—upper panel), and is able to provide immunity however with limited duration.

For the purposes of long lasting anti-tumor humoral immunity, the production of other antibodies isotypes such as IgG is desirable. Physiologically, this usually implies the involvement of Th cells, in a process called T cell-dependent B cell activation (Figure 2—lower panel). Th cells specific for particular peptides are initially activated by antigen presenting cells. Then carbohydrate specific B cells receive stimulatory signals (cytokines) from Th cells, which allow the switching of antibody subtypes from IgM to high-affinity IgGs and the differentiation of plasma cells into memory B cells [47]. These high-affinity IgG antibodies can bind to the target cancer cells, marking them for destruction by either the complement (complement dependent cytotoxicity (CDC)) or by Natural killer (NK) cells (antibody-dependent cell-mediated cytotoxicity (ADCC)).

The knowledge of these physiological mechanisms raised, in recent years, efforts to elicit increased immunogenicity of carbohydrate antigens, aiming to generate potent immunotherapies, such as vaccination. The approaches to improve carbohydrate immunogenicity include the covalently coupling of carbohydrates to immunologically active protein carriers, such as keyhole limpet hemocyanin (KLH) or adjuvants or to physiological/pathological carriers, such as mucin 1 protein (MUC1), to guarantee a range of relevant epitopes [48]. Interestingly, several studies have demonstrated that the conjugation of glycosylated MUC1 peptides with a peptide T helper cell epitope, a Toll-like receptor 2 agonist, tetanus toxoid, or a combination of these active protein carriers induces a potent antigenic and cellular immune response that can kill the tumor cells and reduce the tumor burden [4,49,50,51,52,53].

### Immune Response and Tn/STn Antigens

There are a relevant number of reports describing immune responses against the Tn or STn antigens. For instance, autoantibodies against STn and Tn mucin 1 glycopeptides were identified in breast and colorectal cancer patients [54,55]. These autoantibodies were found at higher levels in early stage breast cancer patients and associated with reduced metastases rate [54], suggesting a role in inducing antitumor immune response. Significantly, these antibodies react with the carbohydrate only when carried on the MUC1 protein, emphasizing the role of the peptide backbone in anti-carbohydrate response. A later study with a higher patient cohort showed that autoantibodies to MUC1 peptides or glycopeptides could not be used for cancer screening [56].

Pre-clinical and clinical studies have also shown that immunization with vaccines containing STn or Tn usually induces antigen specific antibodies [57,58,59]. The elicitation of such immune responses after vaccination could explain the observed delay in tumor growth in mice and the increased time to progression in patients [58,59]. Interestingly, patients receiving the vaccine Theratope, which consisted of STn anchored to the KLH, developed anti-STn antibodies, whose abundance was directly correlated with disease free survival [60]. However, the observed immune responses against STn and Tn antigens, seem to be insufficiently robust to induce protective immune responses. In fact, in spite of the fact that STn and/or Tn-based cancer vaccines have already entered clinical trials (NCT00030823) including the case of Theratope that reached Phase III (NCT00046371) [58], no vaccine has been approved for clinical use yet. Most tested vaccines failed to induce robust T cell-mediated immunity and did not bring any survival benefit for patients. A better understanding of the immune response against these antigens is advised to elicit increased immunogenicity to improve potency of immunotherapeutic approaches.

In recent years, a better understanding of the immunomodulation role of the Tn and STn antigens has elucidated their contribution to immune tolerance, thus explaining in part the failure with vaccination protocols. For example, Tn antigen expressed in mucin 6 (MUC6) protein abrogates Th1 cell responses and promotes interleukin 17 (IL-17) response, which might favor immune escape of tumor cells [61]. Moreover, Tn is recognized by the tolerogenic lectin—macrophage galactose *C*-type lectin (MGL), expressed by DCs and macrophages, which endows these cells to suppress T cell immunity, thus playing a role in tumor progression [62].

The overexpression of STn is associated with low infiltration of nest CD8^+^ lymphocytes in endometrial cancer [63]. Furthermore, STn present in secreted colon cancer mucins can interact with CD22 and down-modulate B cell signal transduction [64]. Moreover, siglec-15 recognizes the STn antigen expressed by cancer cells and transduces a signal for enhanced transforming growth factor beta (TGF-β) secretion in tumor-associated macrophages, which can contribute to tumor progression by the TGF-β-mediated modulation of intratumoral microenvironment [65]. More recently, our group demonstrated that STn-expressing cancer cells impair maturation of DCs, endowing a tolerogenic function and therefore limiting their capacity to trigger protective anti-tumor T cell responses [66]. We have also observed that blockade of STn antigens expressed by cancer cells was able to lower the induction of tolerance *in vitro* and DCs become more mature [66]. These findings suggest that targeted therapies based on antibodies may provide efficient means to enhance immune responses against STn tumor cells.

## 4. Antibodies

For the last 20 years, monoclonal antibody-based treatment has been one of the most successful therapeutic strategies in different fields, including cancer. Due to their unique features, such as high specificity and engagement with the immune system, antibodies’ impact has been recognized in many therapeutic areas [67,68]. The unceasing development and optimization of methods involved in antibodies engineering, production and purification, as well as the increasing knowledge of the interplay between antibodies, cancer cells and immune system have contributed to the development of innovative next generation antibodies which are more effective, safer and with broader applications [69,70,71]. Nowadays, the market for therapeutic antibodies has been growing significantly within the healthcare industry so that 40 years after the generation of the first monoclonal antibody (mAb) [72], around 47 recombinant monoclonal antibody products have been approved in the United States or Europe for the treatment of a wide variety of diseases, ranging from cancer to infectious and cardiovascular diseases to autoimmune diseases [73,74].

### 4.1. Structure and Role of Antibodies

**Figure 3:**
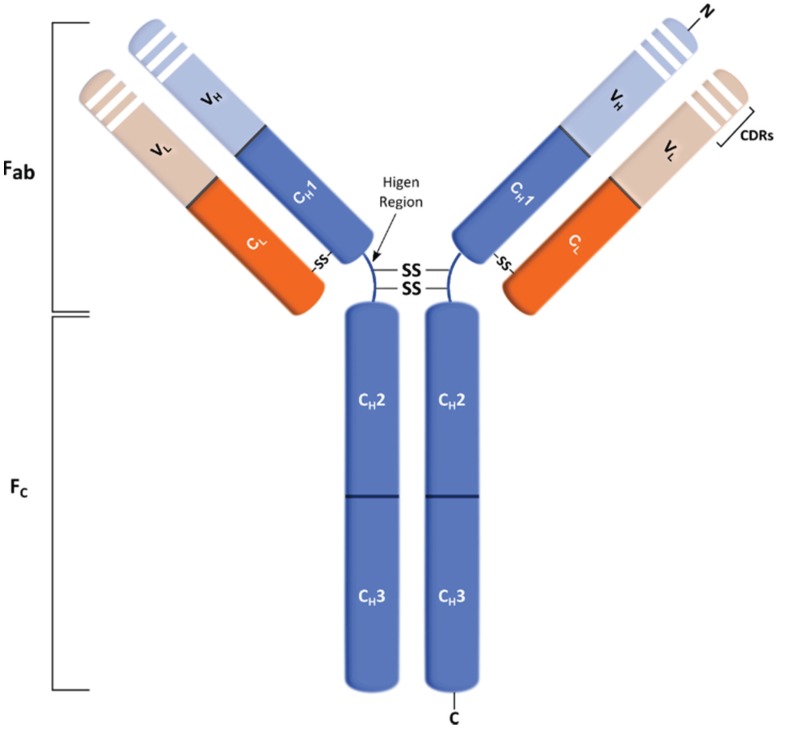
Schematic representation of an immunoglobulin G (IgG) mAb structure. The IgG molecule is composed by constant (C) and variable (V) domains for each light (L) or heavy (H) chain. The heavy chain comprises three constant domains (C_H_) and one variable (V_H_), and the light chain contains one constant (C_L_) and one variable (V_L_) domain. This molecule can be divided into F_c_ and F_ab_ regions in which the latter includes the variable regions, called complementarity-determining regions (CDRs).

Antibodies are glycoprotein molecules with a remarkable ability to recognize and bind to antigens with high affinity and specificity, thus further promoting their inactivation or elimination [68]. Typically, antibodies (or immunoglobulins (Igs)) are composed of two antigen-binding fragments (Fab) linked via a flexible region (hinge region) to a constant (Fc) region (Figure 3). Antigen specificity is conferred by the antigen binding site of the antibody, which is formed by the hypervariable complementarity-determining regions (CDRs) on the variable regions of each heavy and light chain, present in the Fab portions. On the other hand, the Fc region is responsible for immune effector functions of the antibodies, promoting the binding to various effector molecules and cells of the immune system [75,76].

Antibodies’ mechanism of action involves ADCC, CDC, or blockage of the action of specific molecules. Additionally, antibodies can also function as signaling molecules [77]. Specifically, ADCC is an effector mechanism in which antibodies direct NK cells to kill antigen-expressing cells. This mechanism relies on the engagement of Fc receptors expressed by NK cells leading to their activation and exocytosis of the cytolytic granule complex perforin/granzyme, resulting in the destruction of target cells by apoptosis [78]. CDC, also known as the classical pathway of the complement system, is a cytolytic cascade mediated by a series of complement proteins (C1–C9) that are abundantly present in the serum. It starts with the binding of the complement molecule, C1q, to the Fc domain of the antibody (IgG or IgM) bound on the surface of the target cell [77]. This triggers the subsequent complement cascade that leads to the lysis of target cells. Antibodies can also present a blocking activity, which prevents growth factors, cytokines and other soluble mediators from reaching their target receptors [75]. Finally, antibodies can induce the crosslinking of receptors that are connected to mediators of cell apoptosis, such as caspases, leading to cell death [79]. Antibodies that retain these functions represent an important therapeutic strategy in many different fields.

### 4.2. Therapeutic Antibodies in Cancer

Therapeutic antibodies are now one of the most successful and important strategies for treating cancer patients. In their early years, therapeutic antibodies were derived from murine or based on a mouse antibody amino acid sequence, which led to potential issues concerning the generation of human anti-mouse antibodies resulting in immunogenicity or allergic-like reactions. The use of murine antibodies also has the problem of low Fc effector function since murine Fc region are not correctly recognized by human Fc receptors. This results in low efficiency in recruiting the human immune system, including ADCC and CDC, which prevents the effective use of murine antibodies in therapy. In alternative, the next generation of therapeutic antibodies involved genetically engineered fusion of mouse variable with human constant domains, which resulted in a more favorable safety profile and half-life, while retaining the specificity of murine antibodies [80].

Therapeutic antibodies can function through mediating alterations in antigen or receptor functions, modulating the immune system or delivering a specific drug that is conjugated to an antibody that targets a specific antigen. Nowadays, there are molecular techniques that can alter antibody effector function, size and immunogenicity contributing to the development of new and more effective antibody-based therapies [81].

**Figure 4:**
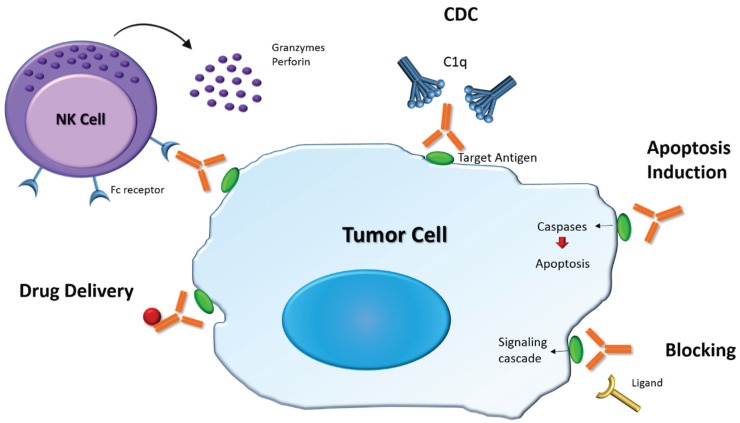
Main functions of therapeutic mAb. mAb can induce antibody dependent cell-mediated cytotoxicity (ADCC) by inducing the release of cytotoxic granules in effector cells (e.g., NK cells). It can also induce complement dependent cytotoxicity (CDC), which starts with the binding of C1q to the antibody triggering the complement cascade. mAb can induce apoptosis as well by activating caspases. In addition, mAb can block receptor/ligand interactions preventing signaling cascade activation, as well as specifically deliver drugs into tumor cells.

Therapeutic antibodies can kill tumor cells by several mechanisms, by direct action of the antibody, which includes: The blocking of receptors or agonist activity; induction of apoptosis or delivery of a drug or cytotoxic agent; or immune-mediated cell killing mechanisms, which involves CDC, ADCC, and induction of phagocytosis (Figure 4). Furthermore, T cells activation can be improved by antibody-mediated targeting and further cross-presentation of antigen to DCs, T cells targeting to the tumor is improved by antibody-mediated blockade of T cell inhibitory check points (e.g., CTLA4 and PD1) are also among the strategies for tumor cell elimination. All of these approaches have been successfully applied in the clinic [82,83,84,85].

Particularly, the target antigen of therapeutic antibodies should be expressed on the surface of cancer cells and be abundant and accessible to antibodies. Depending on the desired mechanisms of action, therapeutic antibodies could be quickly internalized, in case of delivery of toxins into cancer cells and downregulation of cell surface receptors. On the other hand, in ADCC and CDC they should remain on the cell’s surface since the Fc region should be available to immune cells and complement proteins [81].

## 5. Therapeutic Antibodies against Tn and STn Antigens

Due to the association of the Tn and STn antigens with several types of cancers as well as to tumor progression and metastasis, efforts have been made to develop antibodies against these glycosylated structures. In addition, the fact that cancer patients are able to produce autoantibodies, prompted its use as biomarkers for early detection of cancer [86] or response to therapy. Interestingly, antibodies found in cancer patients have not proven to be efficient in activating the immune system and eliminating the malignant cells. Therefore, it is essential to produce more effective antibodies that will specifically kill cancer cells.

So far, the majority of the existent antibodies were obtained by hybridoma technology. Since the development of the first anti-STn specific mAb in 1981, named B72.3 [87], more antibodies against STn antigen have been produced. During this quest, different immunogens and techniques were used and these lead to the development of antibodies exhibiting different affinities and specificities towards the STn antigen [46]. The B72.3 mAb was generated following immunization of mice with membrane-enriched fraction of human breast carcinoma cells and found to react with TAG-72, a glycoprotein with mucin-like characteristics, and with ovine submaxillary mucin (OSM), which is rich in STn epitopes [87,88]. The second generation antibody CC49 was generated following immunization of mice with B72.3 affinity purified TAG-72 molecules [89]. Further studies have been developed using this antibody and its humanized format, which is currently undergoing a clinical trial for its use in radioimmunoguided surgery. However the fine specificity of these mAbs is unclear and in addition to bind to STn, CC49 also recognize other glycans. TKH2 [90] and HB-STn1 are two other anti-STn mAbs that were generated by immunization of mice with OSM [88]. Ogata and colleagues reported that B72.3 strongly binds glycoproteins bearing STn-trimers, but poorly interacts with monomeric-STn glycoproteins [91]. This raised the question of STn configurations for antibody recognition. Later, the antibody MLS102 generated by immunization of mice with LS 180 colonic cancer cells was reported to be specific for clustered STn [92]. More recently, novel anti-STn antibodies LLU9B4 and 3P9 have been produced. The first was generated by immunization of mice with STn glycoproteins extracted from human colon adenocarcinoma cell line LS174T [93] and 3P9 was obtained using human colorectal adenocarcinoma SW1116 cells to immunize mice [94]. This last mentioned antibody is particularly interesting since it is an IgM mAb that showed significant inhibitory effect on proliferation and migration of STn expressing cells and tumor growth by inducing apoptosis. These results suggested that 3P9 mAb has potential applications, not only in diagnosis and imaging, as reported for most of the previous mentioned antibodies, but also for antibody-based tumor therapy [94].

Several mAbs with different specificities towards the Tn antigen have also been generated using different immunogens. Some of these antibodies are CU-1 [95], MLS 128 [96], BRIC 66 (IgM) and BRIC 111 (IgG1) [97], PMH1 [98], and more recently the mAbs KM3413 [99], 2154F12A4 [100] and GOD3-2C4. Monoclonal antibodies, such as MLS128, KM3413 and GOD3-2C4 have been shown to have a therapeutic potential due to their ability to inhibit the growth of certain cancer cell lines [101], and particularly limit the growth of a xenografts of a human lung carcinoma, in the case of the mAb GOD3-2C4 [102].

While most of these antibodies are promising, their specificity is still debatable and the potential therapeutic effect on cancer cells and patient tissues needs to be thoroughly assessed prior to their use as therapeutic agents. A widely used technique to characterize an antibody’s specificity is Enzyme-linked immunosorbent assay (ELISA) and many variations of this methodology against glycoconjugates that are immobilized in plastic or alternatively coating plates with the antibodies. However, in most cases the glycan antigens may not be available and the interpretation is difficult. Therefore, there is still the need to develop assays that fully characterize this specificity of anti-carbohydrate antibodies. Recent efforts in this regard have been accomplished with the development of glycan microarray technologies, which allow the study of interactions between antibodies with carbohydrates in a high-throughput manner [103]. Additionally, existing antibodies are carbohydrate reactive antibodies, meaning that they react with any glycoprotein carrying the glycan. Only a few were able to recognize glycan/peptide epitopes [104] though with low affinity. Antibodies to promising STn or Tn glycopeptide epitopes expressed by cell surface glycoproteins would improve specificity and avoid binding to irrelevant extracellular glycoproteins, such as mucins, which may deceive effective binding of antibodies to cell surface.

### 5.1. Antibody Production

The urge for therapeutic application of antibodies allied to advances and innovation in molecular biology throughout the years allowed for the development of several methods for mAb production. These technologies endorsed the generation of antibodies against virtually any target molecule on human cells allowing their particular employment in cancer therapeutics. Initially, mAbs production was exclusively accomplished using the hybridoma technology [72] and B cell immortalization using Epstein-Barr virus (EBV) [105]. A few years later, the development of alternative methods such as immunization of transgenic mice expressing human Ig loci [106] and phage display [107] enabled further progress with the production of fully human antibodies.

#### 5.1.1. Hybridoma Technology

**Figure 5:**
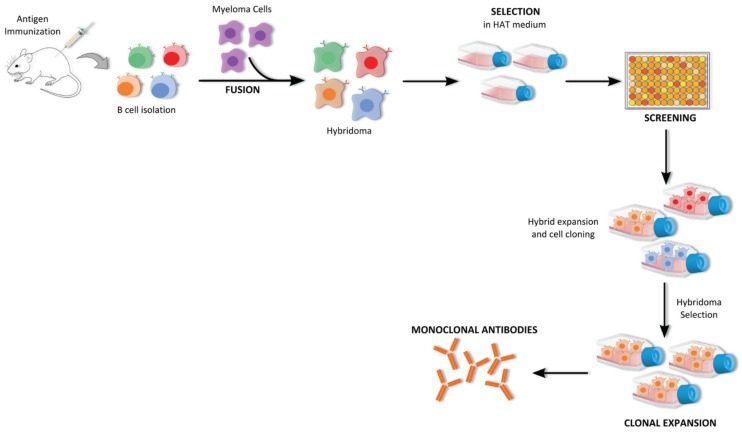
Schematic representation of the hybridoma technology. This technology involves the initial step of mice immunization with desired antigen, followed by the recovery of B cells from mice spleen and consequent fusion with myeloma cells. After that, hybridoma cells are selected in HAT medium and clones are screened according to the reactivity of their supernatants against the antigen. Finally, the selected clones are further cloned and expanded in order to obtain monoclonal antibodies with the desired specificity.

Hybridoma technology has long been a remarkable and indispensable tool for generating high-quality mAbs. It allows the isolation and production of mAb with high affinity and specificity against soluble or membrane-bound antigens using an animal’s immune system [108,109]. This technology was developed in 1975 by Kohler and Milstein [72], who received a Nobel Prize for this achievement, in which the production of antibodies by spleen B cells obtained from immunized mice was immortalized by fusion with cancerous myeloma cells (Figure 5) [110,111]. The initial step in mAbs production by hybridoma technology is stimulation of the immune system of a suitable host, normally a mouse, with the desired antigen. Usually, the antigen is mixed with an adjuvant to enhance the immune response and consequently increase antibody production. After immunization, antibody-secreting B cells are isolated from the mouse spleen and fused, by using polyethylene glycol (PEG) with an immortalized immunoglobulin non-secreting myeloma cell line that lacks the hypoxanthine-guanine-phosphoribosyltransferase (HGPRT) gene. These cells are then cultured in hypoxanthine-aminopterin-thymidine (HAT) selection medium in which only the hybridoma cells are able to survive and proliferate since they have inherited immortality of myeloma cells and selective resistance of B cells to survive in medium with aminopterin. Usually, the screening of the desired antibody-producing cells is performed using techniques such as ELISA, Western Blot and flow cytometry to analyze the reactivity of their culture supernatant. Positive hybridoma cells are further grown and cloned so high amounts of mAbs can thus be produced *in vitro* [74,112,113,114].

Several technical improvements have been accomplished to solve the troublesome of this technique, namely, the immortalization of mice B cells and development of different partner cells for fusion; use of feeder layers to increase hybridoma survival and stability in culture and use of different methods for cell fusion, namely electrofusion [110,115].

The first antibody to be approved for the market, muromonab-CD3, was an unmodified murine antibody developed using the hybridoma technology [116]. However, murine mAbs cause immune reactivity when applied to humans and also have low Fc effector function due to the inefficient recognition of their Fc region by human Fc receptors, thus limiting their applications in therapeutics [74]. Consequently, the use and production of human mAbs was boosted in a way that alternative methods have been studied and developed, such as immunization of transgenic mice expressing human Ig loci, phage display libraries, and humanization of mouse antibodies via genetic engineering [106,117,118].

#### 5.1.2. B Cell Immortalization

Soon after the introduction of the hybridoma technology, an alternative approach to obtain stable human antibody producing B cell lines was created in which antigen-specific plasma B cells are immortalized using the Epstein-Barr virus (EBV) [119]. This virus belongs to the herpes family and has the ability to efficiently immortalize *in vitro* nearly all human B cells. Due to the low frequency of antigen-specific B cells among peripheral blood lymphocytes of donors, the cells expressing the desired antibody on their surface need to be selected. After selection, target specific B cells are infected with EBV. The B cells are transformed into immortalized diploid cells that usually do not release the virus particles and preserve the characteristics of the initially infected B cells, meaning that they are able to continuously produce and secrete the desirable antibodies [120]. The antibodies produced by this method represent the antibody repertoire of the lymphocytes’ donor and, when using a patient’s B cells, it offers the possibility of obtaining antibodies with unique specificity against neoantigens. However, the EBV-transformed cells cannot grow indeterminately since they do not have the phenotype of malignant cells. In addition, these cells are difficult to clone, and usually produce only low yields of immunoglobulins [119,120]. In this way, improvements to this method have been carried out, namely fusion of the EBV-immortalized antibody secreting cells with appropriate heterohybridoma fusion partners, which promoted increased stability and higher amount of secreted antibodies [121]. This technique has been successfully applied to generate antibodies against several antigens, such as proteins and carbohydrates [122,123], but never against STn and Tn.

#### 5.1.3. Phage Display Technology

The main struggle in traditional methods is the development of fully human mAbs to apply in therapeutics and consequently, several efforts have been endeavored to overcome this problem. The debut of recombinant DNA technology together with progress of *in vitro* technologies provided several new and powerful ways that lead to the development of the phage display technology to create therapeutic antibodies. This ground breaking technology was introduced in 1985 by Smith [107] centered on the selection of a particular phenotype (e.g., a ligand specific to a target antigen) from repertoires of molecules displayed on phages. In 1990, McCafferty and colleagues demonstrated that the display of antibody fragments on phages was conceivable through the use of vectors to introduce antibody DNA into phage genomes, thus combining genotype and phenotype in one phage particle [117,124]. The concept behind this display technology is that a large library of potentially interesting antibodies is created, from which antibody fragments with desirable specificity and affinity can be selected against a specific antigen [125]. The use of phage libraries carrying human Ig genes-antibody display libraries was one of the most successful applications of this approach [125].

**Figure 6:**
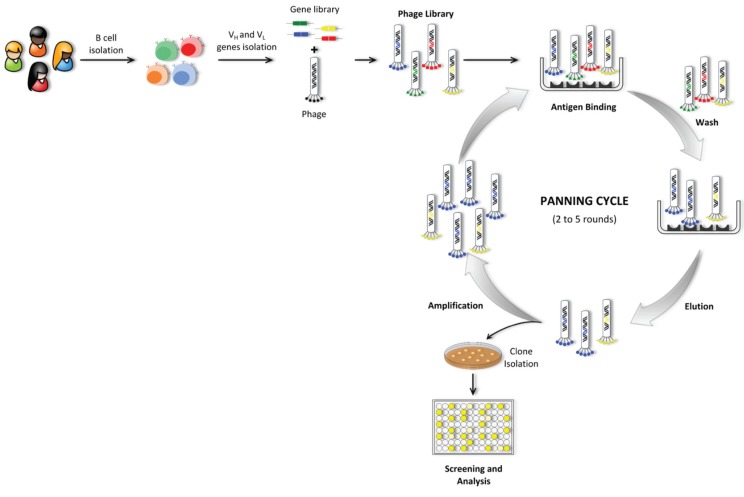
Schematic representation of the phage display technology. This technique includes three main steps that begin with antibody phage library construction and antibody fragments’ display onto the phage surface, followed by several rounds of selection against the target antigen (termed panning cycle) and lastly, clone isolation and subsequent screening for fragments with desired specificity.

For the construction of an antibody library, the repertoire of V-genes encoding antibodies from B cells is amplified via PCR using a set of specific primers covering all the V gene families. Subsequently the antibody fragments are generated by random combination of VL and VH chain genes [126,127]. Based on the origin of antibody genes, the libraries can be categorized into natural or synthetic. Furthermore, natural source repertoire libraries can be either naive or immune. Naive libraries are derived from non-immunized donors of B cells, and immune libraries are derived from different kinds of immunized animals, human patients with a certain disease or previously exposed [75]. Additionally, several formats of antibody fragments, most commonly Fab and scFv can be cloned and displayed on phage libraries [128]. Once created the repertoire of antibody fragments, this heterogeneous mixture of phage clones is then screened by an affinity selection process called panning, during which the phage populations are presented to specific targets in order to select specific target-binding phages (Figure 6). The antigen of interest is immobilized on a solid support and the antibody phage library is applied to promote the binding of specific phages against the selected antigen. Extensive washes are performed to remove unbound phages which do not display specificity against the target antigen. Positive binding phages are then eluted and amplified through bacterial infection and grown to produce high phage titer and use in further rounds of selection. Typically, several rounds of panning are performed (2 to 5 rounds) in order to progressively enrich the phage pool with specific binding phages. Antibodies specific for the desired antigen are usually identified by ELISA by screening a set of random clones that were chosen to be analyzed [125,129].

Once antibody fragments of interest are isolated, due to the link established in phage display between genotype and phenotype, the sequence encoding these molecules can be promptly determined and used to produce the desired antibodies, as well as to refine the isolated antibodies’ properties [125,130]. Currently, there are three antibodies on the market created using the phage display technology Humira [131], Benlysta [132], and AbThrax [133]. Particularly, research using this technology has been making progress on the production of antibodies against challenging antigens, namely tumor associated carbohydrates. In particular, this strategy can be an alternative to conventional hybridoma technology, especially when aiming to obtain antibodies against simple and small conformational antigens, with low immunogenicity [134]. Kubota and colleagues have successfully used the antibody phage display technology to identify an anti-Tn scFv antibody [135]. This was reformatted into a human IgG with promise for therapy, since it has been proven to bind specifically to Tn-positive tumor cells and to induce significant cytotoxic activity in ADCC experiments. Moreover, in comparison with wild-type IgG molecules, the scFv-Fc format has potential benefits for therapeutic development and use. These types of antibodies have small molecular weight and therefore are thought to have better tissue penetration. In addition, since they are composed of a single polypeptide chain, heteromeric disulfide association is not required and consequently, expression of functional scFv-Fcs has been reported using yeast [135].

#### 5.1.4. Transgenic Mice

Among the several techniques developed to produce human mAbs, recently a technology focused its efforts on the generation of low immunogenic mAbs using transgenic mice expressing repertoires of human antibody gene sequences. The production of human mAbs using the hybridoma technology and genetically engineered mice expressing human antibody repertoires was first reported in 1994 [136]. The engineered mice were generated through the disruption of the endogenous mouse Ig-heavy chain and Igκ-light chain loci to avoid the expression of mouse heavy or light chains. Simultaneously, in order to replace the mouse chains, transgenes encoding human Ig-heavy chain and Igκ-light chain were introduced. Upon immunization, these knockout/knockin mice were able to produce human antibodies and using conventional hybridoma technology, the spleens were further used in the production of human mAbs by hybridoma cell lines [136].

Over the past years, progress has been accomplished and additional V gene segments can be expressed by transgenic mice expanding the repertoire of the antibodies obtained. Currently, several different versions of similarly engineered mice have been developed and exploited by different biotechnological and pharmaceutical companies. In fact, over 50 human mAbs developed using this technology are in clinical trials and by 2010 six indeed reached the market [119].

#### 5.1.5. Molecularly Engineered Antibodies

The expansion of therapeutic antibodies is a dynamic field that evolves alongside with technological developments. Advances in antibody engineering allowed to overcome the main problems associated with the immune response developed against foreign antibodies introduced into humans [137]. Antibody chimerization, humanization and the development of human antibodies were the major strategies adopted to reduce immunogenicity [68]. Additionally, efforts to improve affinity, pharmacokinetics and avidity of therapeutic antibodies as well as enhance their natural effector functions have been employed. Several approaches can be used to manipulate the variable regions in order to increase the binding affinity for antigens, or tune the binding specificity, namely chain-shuffling, randomization of CDRs and induced mutations in the variable regions [138,139]. On the other hand, modifications in glycosylation or amino acid sequences in the constant region can modulate the Fc effector functions of antibodies, since this region is involved in ADCC and CDC [137,139]. Extension of the IgG half-life in serum is accomplished by engineering the Fc region particularly in modulation of its interaction with the neonatal Fc receptor (FcRn) which is associated with immunoglobulin protection from intracellular degradation [140]. Currently, antibody-drug conjugates, multimerization of antibody fragments and production of a variety of formats not found in nature, such as Fab and scFv fragments, diabodies and minibodies are rapidly emerging as key strategies in the antibody engineering field [141,142]. As anticipated, antibody engineering has been fostering continuous research and developments in the refinement and manufacturing of innovative antibodies that display new and enhanced properties to stimulate the antitumor immune response and be applied in therapeutics.

## 6. Conclusions

Antibodies against STn and Tn antigens have initially raised a great interest due to their potential use as diagnostic tools. This interest was further extended to the exploitation of the antibodies’ predictive value in vaccinated patients. Unexpectedly, there has been no (or very limited) efforts in extending the therapeutic use of antibodies to boost antitumor immune responses or conjugated to toxic payloads. The fact that no therapeutic antibodies have entered into clinical trials could be explained by an apparent lack of interest given the glaring promise of vaccination or by an interlude while new antibodies are being developed by academic or biopharmaceutical companies. Undeniably, there is the need to develop novel antibodies with improved specificities targeting STn or Tn. Alternatively, antibodies against glycopeptides could be a suitable approach to increase their specificity. Obtaining antibodies with such specificity for the glycan conformation is technically demanding, therefore novel cutting-edge methodologies herein described emerge as very attractive resources to accelerate the development of therapeutic anti-Tn and -STn antibodies that could be particularly useful in enhancing immune responses or as drug delivery systems.
